# Performance Enhancement of an Acoustic Energy Harvester with a Flexible Polyvinylidene Fluoride-Based Piezoelectric Nanogenerator via the Thermoacoustic Effect

**DOI:** 10.3390/nano16140848

**Published:** 2026-07-10

**Authors:** Liu Liu, Geng Chen

**Affiliations:** 1 Zhejiang Key Laboratory of Intelligent Manufacturing for Aerodynamic Equipment, College of Mechanical Engineering, Quzhou University, Quzhou 324000, China; 36117@qzc.edu.cn; 2 National Engineering Research Center of Power Generation Control and Safety, School of Energy and Environment, Southeast University, Nanjing 210096, China

**Keywords:** acoustic resonator, thermoacoustic effect, polyvinylidene fluoride-based piezoelectric nanogenerator, temperature difference, acoustic energy harvesting

## Abstract

This study proposes a novel approach for performance improvement of an acoustic energy harvester integrated with a flexible polyvinylidene fluoride-based piezoelectric nanogenerator by exploiting the thermoacoustic effect. A prototype of the acoustic energy harvester was first designed and constructed. The influence of the temperature difference across the thermoacoustic stack on the acoustic pressure and open-circuit voltage amplitudes was then experimentally examined. Subsequently, the effects of excitation frequency and driving voltage on the performance of the acoustic energy harvester were systematically analyzed. The results demonstrate that the thermoacoustic effect can be effectively employed to enhance acoustic oscillations and, consequently, improve the electrical output. As the excitation frequency changes, the acoustic oscillations inside the acoustic energy harvester and the open-circuit voltage of the piezoelectric nanogenerator can be either amplified or suppressed depending on the frequency range. In addition, optimal driving voltages exist at which the amplification of acoustic pressure and open-circuit voltage is maximized. Specifically, at an excitation frequency of 85 Hz, a driving voltage of 3.5 V, and a stack temperature difference of 101.5 °C, a maximum pressure amplification factor of 3.73 and a maximum voltage amplification factor of 1.15 are obtained. This study successfully demonstrates the feasibility of utilizing the thermoacoustic effect to amplify acoustic oscillations within a resonator, offering a new pathway for enhancing the performance of acoustic energy harvesters and expanding the application potential of thermoacoustic technology.

## 1. Introduction

Acoustic energy harvesting, as an emerging approach for exploiting ambient energy, has attracted considerable attention due to its potential applications in low-power sensors, self-powered monitoring systems, and Internet of Things (IoT) nodes [1]. Acoustic energy is ubiquitously present in industrial machinery, transportation systems, and environmental noise, and is characterized by relatively stable availability and flexible harvesting configurations [2]. The fundamental principle of acoustic energy harvesting lies in converting ambient sound pressure oscillations into usable electrical energy, typically by inducing structural vibrations or fluid motion through an acoustic field and subsequently transforming the mechanical energy into electrical energy via appropriate transducers. At present, the dominant acoustic energy harvesting strategies include those based on piezoelectric effects [3], electromechanical–electromagnetic coupling [4], and triboelectric mechanisms [5]. Piezoelectric harvesters are compact and highly sensitive, electromagnetic devices are well suited for low-frequency and large-displacement conditions, while triboelectric harvesters exhibit significant potential for low-frequency and broadband acoustic energy harvesting.

The performance of acoustic energy harvesters can be enhanced through several approaches. First, optimization of acoustic structures is critical [6]. The incorporation of resonant cavities, Helmholtz resonators, waveguides, or acoustic metamaterials can effectively amplify sound pressure levels within targeted frequency bands, thereby increasing acoustic energy density. Concurrently, appropriate design of cavity geometry and boundary conditions enables frequency matching and modal reinforcement. Second, aligning the natural frequencies of the system with dominant acoustic frequencies is essential [7]. The adoption of multimodal configurations, tunable mechanisms, or nonlinear designs can significantly broaden the operational bandwidth and improve adaptability to complex acoustic environments. Third, the transducer performance can be further improved by enhancing electromechanical coupling through optimized piezoelectric materials, electromagnetic coil designs, or triboelectric structural parameters [8]. In addition, careful selection of materials, structural amplification schemes, and polarization strategies contributes to higher energy conversion efficiency. Finally, effective impedance and load matching (achieved through the optimization of electrical loads, rectification circuits, and energy storage units, which facilitates multilevel acoustic–mechanical–electrical impedance matching) will minimize energy reflection and dissipation [9].

Beyond these conventional strategies, thermoacoustic effects provide an alternative and promising pathway for enhancing acoustic energy harvesting performance [10]. The thermoacoustic effect arises from the interaction between an oscillating fluid and a solid boundary subjected to a temperature gradient [11,12,13]. When a temperature difference exists along the solid surface, the oscillating fluid undergoes a clockwise thermodynamic cycle during fluid–solid interaction, absorbing thermal energy and converting it into acoustic power [14]. Traditional thermoacoustic research has focused on the development of thermoacoustic engines, in which porous media are employed to increase fluid–solid contact area and enhance acoustic power output [15,16,17]. Depending on the acoustic boundary conditions, standing-wave [18] and traveling-wave [19] thermoacoustic engines can be constructed to achieve efficient thermal-to-acoustic energy conversion. These engines can be further integrated with acoustic–electric transducers, such as linear alternators [20], piezoelectric elements [21], or triboelectric nanogenerators [22], to realize the conversion of amplified acoustic energy into electrical energy. Owing to their simple structure, absence of moving mechanical parts, high reliability, and environmentally benign working fluids, thermoacoustic engines and generators exhibit broad prospects in thermal energy harvesting [23,24].

Although substantial progress has been made in thermoacoustic research with an emphasis on thermodynamic performance metrics such as output power [25] and thermal efficiency [26], relatively limited attention has been paid to the application potential of thermoacoustic effects from acoustic and vibrational perspectives. To address this gap, the present study innovatively employs the thermoacoustic effect to enhance the acoustic power and electrical output of an acoustic energy harvester. Experimental investigations are conducted to validate this concept, and a systematic parametric analysis is performed to elucidate the key factors governing acoustic power amplification and electrical performance. The remainder of this paper is organized as follows: Section 2 introduces the experimental prototype of the acoustic energy harvester; Section 3 presents the forced response characteristics; Section 4 describes the thermoacoustic amplification phenomenon; Section 5 examines the effects of excitation frequency and driving voltage on thermoacoustic amplification; Finally, the main conclusions of this study are summarized in Section 6.

## 2. Experimental Study

### 2.1. Configuration of the Acoustic Energy Harvester with Thermoacoustic Core

**Figure 1:**
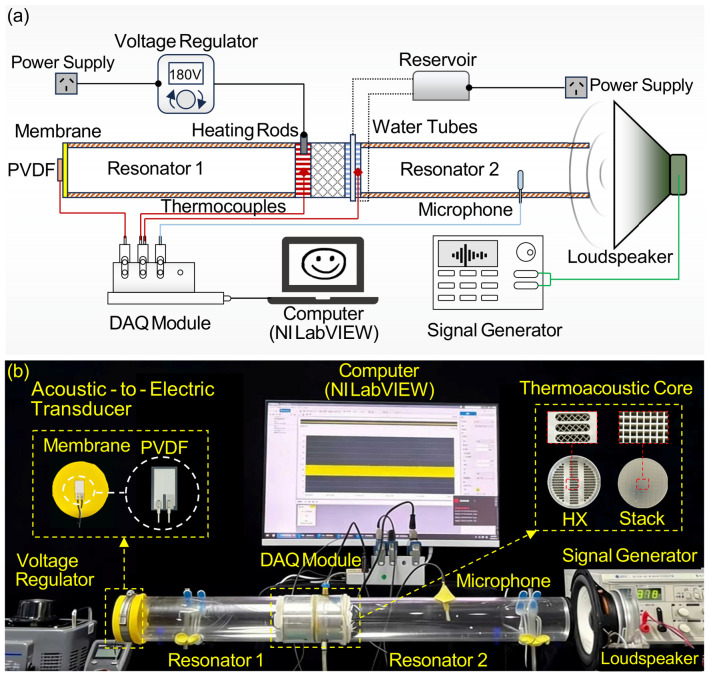
(**a**) Schematic diagram and (**b**) photo of the experimental setup.

Figure 1 illustrates the experimental setup employed in this study. The experimental setup consists of two main components: an acoustic energy harvester with thermoacoustic core and an external loudspeaker. As shown in Figure 1a, the acoustic energy harvester can be further divided into four sections (from left to right): the acoustic-to-electric transducer, acoustic resonator 1, the thermoacoustic core, and acoustic resonator 2. The acoustic-to-electric transducer is composed of a flexible polyvinylidene fluoride (PVDF)-based piezoelectric nanogenerator (discussed later in Section 2.2) mounted on an elastic membrane.

The left end of acoustic resonator 1 is sealed by the elastic membrane made of latex, which is stretched to maintain tension and fixed in place using a clamp. The right end of acoustic resonator 1 is connected to the thermoacoustic core, which consists of a hot heat exchanger, a stack, and a cold heat exchanger. The custom-designed hot and cold heat exchangers comprise horizontally arranged parallel plates that allow air to flow through, as well as vertically oriented circular channels for connection with electric heating rods and water-cooling tubes, respectively, as illustrated in the enlarged view in Figure 1b. The stack, featuring a honeycomb structure (see the enlarged view), is sandwiched between the hot and cold heat exchangers. When the hot heat exchanger is heated by the electric heating rods connected to a voltage regulator and the cold heat exchanger is cooled by circulating chilled water from a reservoir, a stable temperature gradient is established along the stack. The right end of the thermoacoustic core is connected to acoustic resonator 2, whose right end is open. The main structural parameters of the acoustic energy harvester are summarized in Table 1.

In this study, an external loudspeaker (model Z-6.5, sensitivity 88 dB, impedance 4 Ω, rated power 120 W) is employed to produce excitation acoustic waves for the acoustic energy harvester. It is coaxially aligned with acoustic resonator 2 and positioned at the open end with an axial separation of 15 mm. The loudspeaker is driven by a signal generator (model DG1022), which produces sinusoidal signals with adjustable voltage amplitude and frequency. Owing to the acoustic boundary conditions (closed left end and open right end), a standing wave is established within the acoustic energy harvester, with a pressure antinode located at the left end and a pressure node at the right end. The acoustic pressure oscillations induce periodic deformation of the elastic membrane, as well as of the PVDF-based piezoelectric nanogenerator bonded to its surface.

### 2.2. Working Principles

#### 2.2.1. Thermoacoustic Effect

**Figure 2:**
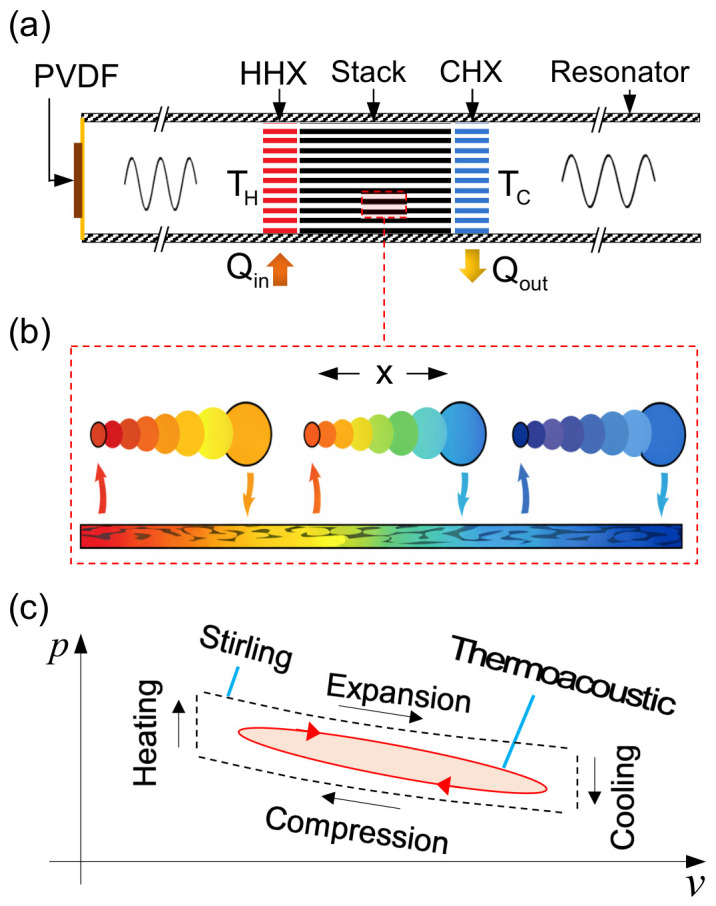
Thermodynamic viewpoint of the thermoacoustic effect. (**a**) Schematic diagram. (**b**) Enlarged view of the fluid parcels near the solid plate. (**c**) Thermodynamic cycles.

This study employs the thermoacoustic effect to amplify pressure oscillations within an acoustic energy harvester. Figure 2a presents a schematic of the acoustic resonator integrated with the thermoacoustic core. The thermoacoustic core is composed of a hot heat exchanger (HHX), a stack consisting of parallel plates, and a cold heat exchanger (CHX). The HHX is heated by heating rods with a heat input of *Q_in_*, and maintains a hot-end temperature *T_H_*, whereas the CHX is cooled by circulating chilled water at flow rate of *Q_out_*, and maintains a cold-end temperature *T_C_*. Consequently, a temperature gradient is established along the axial direction of the stack plates.

Figure 2b presents an enlarged view illustrating the motion of fluid parcels between the stack plates. Under loudspeaker excitation, the fluid parcels within the stack channels undergo periodic oscillatory motion. Since the working fluid (air) is a compressible ideal gas, its temperature increases during compression (leftward motion) and decreases during expansion (rightward motion). If the temperature gradient is sufficiently large, when a fluid parcel moves leftward (toward the hot end), the temperature increase of the neighboring solid plate exceeds the temperature rise of the fluid parcel due to compression. During this process, the fluid parcel extracts heat from the solid plate. Conversely, when the fluid parcel moves rightward (toward the cold end), the temperature decrease of the neighboring solid plate exceeds the temperature drop of the fluid parcel due to expansion. During this process, the fluid parcel releases heat to the solid plate. Consequently, as the fluid parcel moves back and forth, it experiences clockwise thermodynamic cycles in the pressure-specific volume (*p*-*v*) diagram, as shown in Figure 2c. Note that the thermoacoustic cycle resembles that of a Stirling cycle which comprises isothermal compression, isochoric heating, isothermal expansion, and isochoric cooling processes. Since the Stirling-like thermoacoustic cycle proceeds clockwise, positive net work is produced and thermal energy is converted into acoustic energy. As a result, the pressure oscillations are amplified within the resonant cavity.

#### 2.2.2. Flexible Polyvinylidene Fluoride-Based Piezoelectric Nanogenerator

In the present study, a commercial PVDF-based piezoelectric nanogenerator (Model: LDT0-028K; Measurement Specialties, Inc., Hampton, VA, USA) is bonded to an elastic membrane at the left end of an acoustic energy harvester, where it serves as a transducer to convert acoustically induced membrane vibrations into electrical energy. The flexible PVDF-based piezoelectric nanogenerators have attracted significant attention for energy harvesting applications due to their ultrathin structure and high mechanical flexibility, which enable efficient integration onto surfaces with complex geometries [27,28,29].

**Figure 3:**
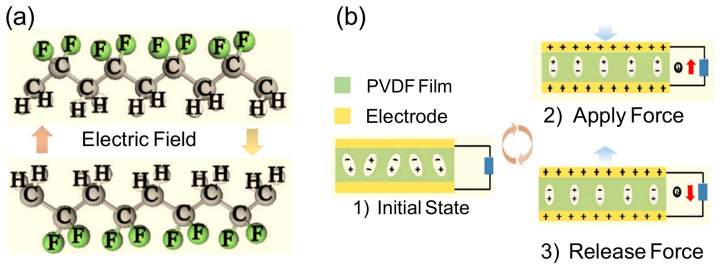
(**a**) Chemical structure of PVDF. (**b**) Working principle of the PVDF-based piezoelectric nanogenerator.

The PVDF-based piezoelectric nanogenerator employed in the present study consists of a 28 μm thick piezopolymer film with (CH_2_–CF_2_) as its repeat unit [30], as shown in Figure 3a. The film has screen-printed silver-ink electrodes, laminated onto a 0.125 mm-thick polyester substrate and equipped with two crimped electrical contacts. Owing to the offset position of the piezoelectric layer relative to the mechanical neutral axis, membrane bending induces substantial mechanical strain within the piezopolymer, thereby enabling efficient electromechanical coupling and the generation of relatively high output voltages. Under dynamic deformation, the PVDF element operates as a flexible switching-type transducer, and the resulting electrical signal is sufficiently strong to directly drive low-power electronic components, such as MOSFET or CMOS circuits.

The working principle of the PVDF-based piezoelectric nanogenerator is illustrated in Figure 3b. In the initial state, when no external mechanical force is applied, the PVDF-based piezoelectric nanogenerator produces no electrical output due to the random orientation of electric dipoles within the piezoelectric film. Upon application of an external force, the film undergoes mechanical deformation, leading to dipole alignment and polarization within the PVDF layer. As a consequence of this polarization process, opposite charges are induced on the two electrodes, resulting in charge transfer from the bottom electrode to the top electrode through the external circuit. When the applied force is released, the compressive stress in the film gradually diminishes, causing the dipoles to relax toward their original unpolarized state. This relaxation process reverses the direction of charge flow until the nanogenerator returns to its initial equilibrium condition. Repeated mechanical loading and unloading therefore produce alternating positive and negative electrical potentials at the output of the nanogenerator [31].

**Figure 4:**
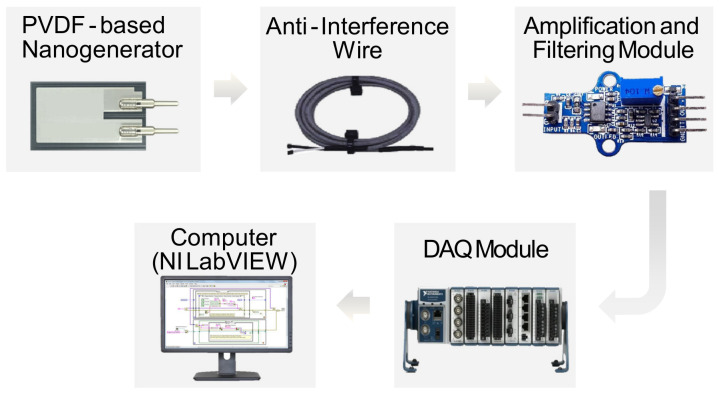
Data collection of the PVDF-based piezoelectric nanogenerator.

In this study, the electrical signal generated by PVDF-based piezoelectric nanogenerator is subsequently amplified using an amplification and filtering module (Model: PVA 103; VKinging, Shenzhen, China [32]), as shown in Figure 4. The acoustic module features a saturated output level of VCC − 0.7 V (upper rail) and GND + 0.7 V (lower rail). The amplification gain can be adjusted via an onboard variable resistor, with a tunable range of approximately 1–100. Under a 5 V power supply, the amplification module outputs a steady voltage level of approximately 2 V in the absence of vibration, while producing an alternating voltage signal with positive and negative fluctuations when vibration is present. Since the charge-type input of the amplifier is inherently susceptible to interference from ambient 50 Hz power-line noise, an anti-interference cable is employed between the PVDF-based piezoelectric nanogenerator and the amplifier to mitigate electromagnetic noise. Finally, the amplified electrical signal is acquired using a C-series voltage input module (NI 9202) and subsequently processed and analyzed on a PC integrated with LabVIEW program.

#### 2.2.3. Measurements and Tests

To characterize the dynamic behavior of the acoustic energy harvester, the pressure, temperature, and electrical signals were measured. An acoustic microphone was positioned at the midsection of acoustic resonator 2 (see Figure 1) to monitor the internal acoustic pressure fluctuations. Two K-type thermocouples were attached to the outer surfaces of the hot and cold heat exchangers to measure their respective temperatures. The piezoelectric PVDF nanocomposite film mounted on the elastic membrane was employed to generate electrical output from the membrane vibration induced by acoustic pressure oscillations. The acoustic pressure and temperature signals, measured by the microphone and thermocouples respectively, were acquired using National Instruments (NI) data acquisition (DAQ) modules, including a C Series temperature input module (NI 9212) and a C Series sound and vibration input module (NI 9230).

The present experimental investigation consists of three tests. Test 1: Forced response of the acoustic energy harvester. In this test, a frequency-sweep excitation is applied to the acoustic energy harvester to obtain its forced acoustic response. During this process, the temperature difference across the thermoacoustic core is maintained at zero, such that the entire system remains at ambient temperature, ensuring that the experiment is purely acoustic in nature. The signal generator outputs a time-domain sinusoidal signal with a constant voltage amplitude of 3.5 V (rms). The excitation frequency is swept logarithmically from *f_start_* = 20 Hz to *f_end_* = 500 Hz, following the time-dependent relation *f*(*t*) = *f_start_* (*f_end_*_/_*f_start_*)*^t/T^*^1^, where *T*1 = 4.32 s denotes the sweep period. Resonance frequencies of the acoustic energy harvester are identified through analysis of the measured acoustic pressure signals. Test 2: Evidence of the thermoacoustic amplification effect. In this test, the excitation frequency and amplitude of the loudspeaker are fixed, while a temperature gradient is imposed across the thermoacoustic core. The resulting amplification of pressure oscillations is monitored to evaluate the contribution of thermoacoustic effects to acoustic energy enhancement. Test 3: Influence of the external loudspeaker. In this test, the hot heat exchanger is heated by heating rods with a constant heat input, and the cold heat exchanger is cooled by the circulating water with a constant water flow rate. The amplitude and frequency of the loudspeaker excitation are varied to explore their effects on the thermoacoustic amplification.

## 3. Forced Responses of the Acoustic Energy Harvester

**Figure 5:**
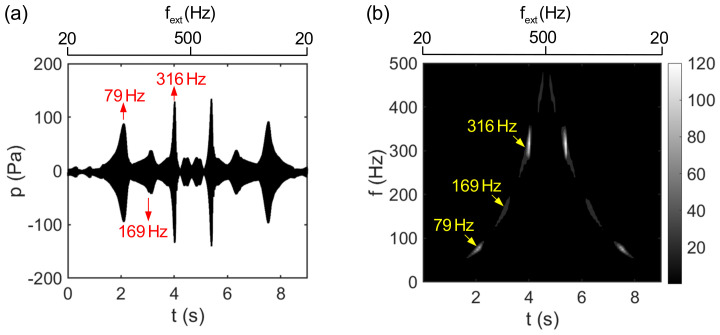
Frequency response of the acoustic energy harvester. (**a**) Acoustic pressure signal. (**b**) Continuous wavelet transform. In the experiment, the temperature difference across the stack is zero.

Figure 5 presents the time history of acoustic pressure and the corresponding continuous wavelet transform (CWT) under frequency-sweep excitation. Figure 5a shows the pressure signal in the time domain during the sweep excitation. As the external excitation frequency *f_ext_* varies with time from 20 Hz to 500 Hz within the first 4.32 s and subsequently from 500 Hz back to 20 Hz during the following 4.32 s, the acoustic pressure exhibits pronounced non-stationary behavior. At specific time instants, the pressure amplitude increases significantly, indicating strong forced responses at certain characteristic frequencies. Notable peaks are observed at approximately 79 Hz, 169 Hz, and 316 Hz, suggesting that when the excitation frequency approaches the resonance frequencies during the sweep, the acoustic pressure amplitude is markedly enhanced. To investigate the impact of the loudspeaker, a standalone frequency response test of the loudspeaker is conducted and presented in Appendix A. Comparison between the frequency responses of the acoustic energy harvester and loudspeaker indicates that the dynamic behavior of the acoustic energy harvester is insensitive to fluctuations in the input sound pressure level of the loudspeaker. The distinct peaks at 79 Hz, 169 Hz, and 316 Hz are identified as the inherent resonant frequencies of the acoustic energy harvester.

Figure 5b displays the corresponding time–frequency spectrum obtained via CWT. The frequency range along the vertical axis is consistent with that of the sweep excitation, and distinct energy concentrations can be observed at specific frequency bands as time progresses. In particular, localized high-energy regions appear near 79 Hz, 169 Hz, and 316 Hz, confirming that these frequencies correspond to the dominant acoustic modes of the system, in agreement with the time-domain observations. The wavelet spectrum clearly demonstrates that multiple acoustic modes are sequentially excited under frequency-sweep excitation. The consistency between the time-domain and time–frequency analyses indicates that the frequency-sweep test effectively identifies the characteristic frequencies of the acoustic energy harvester, thereby providing a reliable basis for subsequent acoustic amplification investigations.

**Figure 6:**
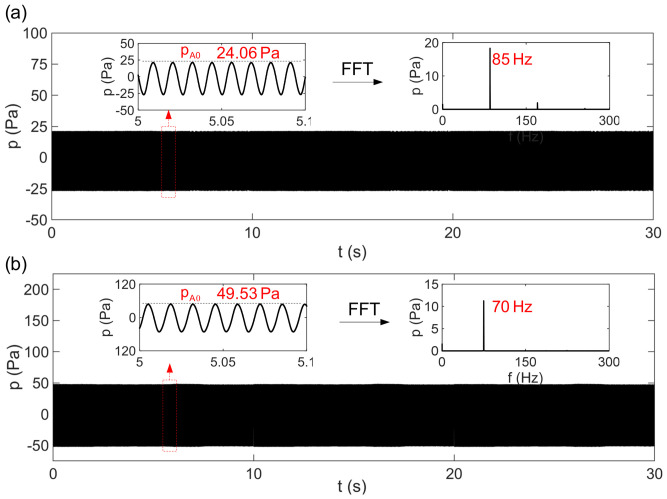
(**a**) Time-domain plot of acoustic pressure and FFT at *V_ext_* = 3.5 V and *f_ext_* = 85 Hz. (**b**) Time-domain plot of acoustic pressure and FFT at *V_ext_* = 3.5 V and *f_ext_* = 70 Hz. In the experiment, the temperature difference across the stack is zero.

Figure 6 further presents the time-domain acoustic pressure and their corresponding frequency-domain analyses under different excitation frequencies. When *V_ext_* = 3.5 V and *f_ext_* = 85 Hz, the system exhibits stable acoustic pressure oscillations with a constant amplitude as shown in Figure 6a. The enlarged view indicates that the pressure waveform is approximately sinusoidal, with an amplitude *p_A_*_0_ of 24.02 Pa. The fast Fourier transform (FFT) reveals a single dominant peak at 85 Hz, while other frequency components are negligible, indicating that the system response is dominated by the driving frequency and that nonlinear harmonics are weak.

Figure 6b shows the results when *V_ext_* = 3.5 V and *f_ext_* = 70 Hz. Compared with the 85 Hz case, the system likewise exhibits periodic oscillations. However, the acoustic pressure amplitude is significantly higher (*p_A_*_0_ increases to 49.53 Pa), indicating that the system possesses stronger acoustic energy near 70 Hz. The corresponding FFT spectrum shows that the spectral energy is highly concentrated at 70 Hz, with no pronounced higher-order harmonics, which further confirms that the system remains in a quasi-linear oscillatory state under this condition. A comparison of the two operating conditions in Figure 6 demonstrates that, at a fixed driving voltage, the acoustic response of the system is highly sensitive to the excitation frequency. The higher pressure amplitude observed at 70 Hz suggests that this frequency is closer to a dominant acoustic mode or resonance frequency of the system.

## 4. Amplification of Acoustic Oscillations Inside the Acoustic Energy Harvester Integrated with the PVDF-Based Piezoelectric Nanogenerator

**Figure 7:**
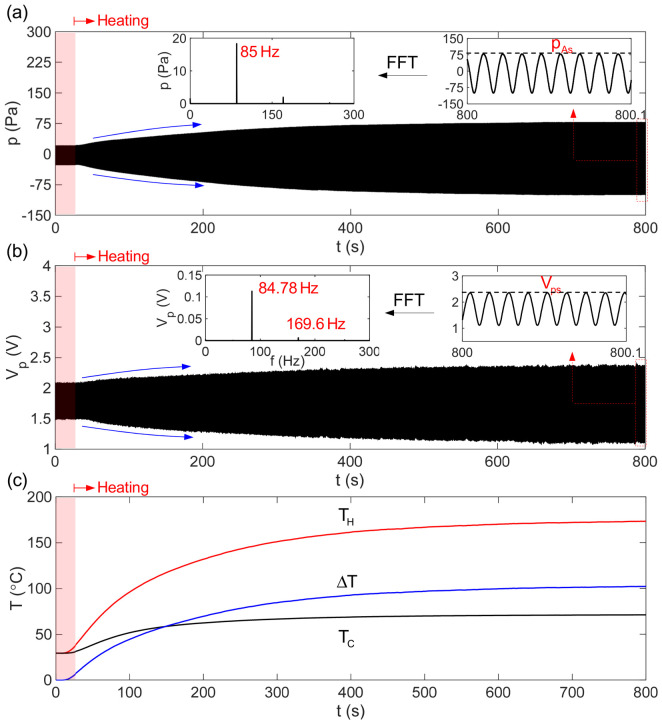
(**a**) Time-domain plot of acoustic pressure and FFT spectrum. (**b**) Time-domain plot of open-circuit voltage and FFT spectrum. (**c**) Temperature curves of hot and cold heat exchangers. In the experiment, *V_ext_* = 3.5 V and *f_ext_* = 85 Hz. The hot heat exchanger is heated by heating rods, and the cold heat exchanger is cooled by the circulating water.

Figure 7 illustrates the influence of the temperature difference across the stack on the acoustic oscillations within the acoustic energy harvester. The time evolution of the acoustic pressure at the open end and its corresponding frequency spectrum is shown in Figure 7a. During the initial stage (0–25 s, indicated by the red shaded region), the system operates under *V_ext_* = 3.5 V and *f_ext_* = 85 Hz with zero temperature difference across the stack. Under this purely acoustic condition, the system response is identical to that shown in Figure 6a, and stable periodic oscillations with a pressure amplitude of *p_A_*_0_ = 24.02 Pa is observed. At *t* = 25 s, electrical power is supplied to the heating rods (*Q_in_* = 35.5 W), and the circulating water pump (*Q_out_* = 800 L/H) is activated. Consequently, the temperature of the hot heat exchanger *T_H_* increases rapidly, while the temperature of the cold heat exchanger *T_C_* rises more slowly, leading to a progressively increasing temperature difference Δ*T*, as shown in Figure 7c.

As Δ*T* increases, the acoustic pressure amplitude gradually rises, and a new steady state is reached after approximately 800 s, corresponding to a temperature difference of about 101.5 °C. The enlarged view in Figure 7a indicates that the steady-state pressure signal remains nearly sinusoidal, with an amplitude of *p_As_* = 89.74 Pa. The corresponding FFT spectrum shows that the acoustic energy is highly concentrated at 85 Hz, coinciding with the excitation frequency, and no pronounced higher-order harmonics are observed. Figure 7b presents the time-domain evolution and frequency-domain characteristics of the PVDF open-circuit voltage measured at the closed end of the resonator. Overall, the open-circuit voltage response follows trends similar to those of the acoustic pressure and temperature difference. As the acoustic amplitude increases, the voltage amplitude rises synchronously from an initial value of 1.8 V and eventually stabilizes at approximately 2.07 V. The FFT result reveals a dominant peak at 84.78 Hz and a secondary harmonic component at 169.6 Hz, indicating the presence of weak nonlinear effects introduced during the energy conversion process, while the response remains dominated by the excitation frequency.

Figure 7 shows that the introduction of a temperature difference across the stack significantly amplifies both the acoustic pressure and the open-circuit voltage, demonstrating that the thermoacoustic effect can be effectively exploited to enhance acoustic power and thereby improve acoustic energy harvesting performance. By defining the pressure amplification factor as *R_p_* = *p_As_*/*p_A_*_0_ and the voltage amplification factor as *R_V_* = *V_ps_*/*V_p_*_0_, the operating condition shown in Figure 7 yields *R_p_* = 3.73 and *R_V_* = 1.15. The discrepancy between *R_p_* and *R_V_* indicates that the voltage output does not scale linearly with pressure amplitude. This implies pronounced nonlinearities in the acoustic–electric conversion system (PVDF nanocomposite film mounted on the elastic membrane), including nonlinear mechanical losses and charge dissipation, which warrant further investigation.

## 5. Impact of the External Loudspeaker

### 5.1. Effect of Excitation Frequency

**Figure 8:**
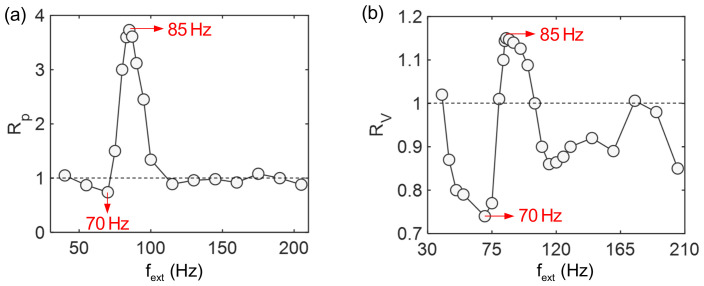
(**a**) Dependence of acoustic pressure amplification factor *R_p_* on excitation frequency *f_ext_*. (**b**) Dependence of open-circuit voltage amplification factor *R_V_* on excitation frequency *f_ext_*. In the experiment, *V_ext_* = 3.5 V. The hot heat exchanger is heated by heating rods, and the cold heat exchanger is cooled by the circulating water.

Building upon the acoustic amplification phenomenon discussed in Section 4, this section further investigates the effects of excitation frequency and driving voltage on the acoustic amplification. Figure 8a shows the variation in acoustic pressure amplification factor *R_p_* with excitation frequency *f_ext_* under the conditions of *V_ext_* = 3.5 V, *Q_in_* = 35.5 W and *Q_out_* = 800 L/H. When *R_p_* > 1, the acoustic oscillations are amplified as compared with the case without a temperature difference. Conversely, *R_p_* < 1 indicates suppression of acoustic oscillations. It can be observed that *R_p_* remains close to unity over most of the investigated frequency range. However, a prominent peak with a maximum value of 3.73 appears near 85 Hz, indicating the strongest amplification of acoustic oscillations. In contrast, *R_p_* with a minimum value of 0.8 is observed near 70 Hz, implying the strongest suppression of acoustic oscillations.

Figure 8b presents the dependence of the voltage amplification factor *R_V_* on the excitation frequency *f_ext_*. Although the overall trend of *R_V_* is similar to that of *R_p_*, noticeable differences exist in both magnitude and frequency range. *R_V_* reaches a maximum value of 1.15 near 85 Hz, indicating that the open-circuit voltage is effectively enhanced and that the acoustic-to-electric energy conversion is efficient. By contrast, *R_V_* is significantly lower than unity near 70 Hz, reflecting reduced electrical output and weak acoustic-to-electric energy conversion. It is worth noting that in certain frequency ranges, *R_p_* is close to or slightly greater than unity while *R_V_* remains below unity. This discrepancy suggests that the electrical response is not solely governed by the acoustic pressure amplitude but is also strongly influenced by the frequency response characteristics of the PVDF-based piezoelectric nanogenerator and the impedance matching of the overall system.

### 5.2. Effect of Driving Voltage

**Figure 9:**
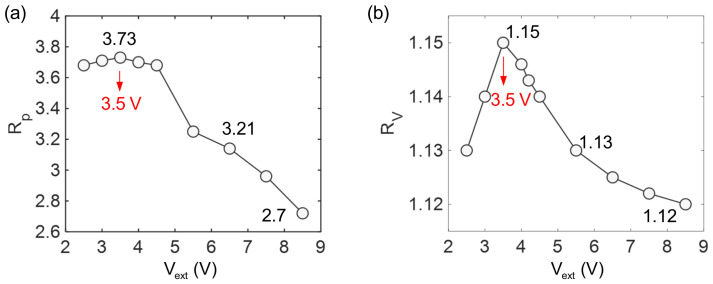
(**a**) Dependence of acoustic pressure amplification factor *R_p_* on driving voltage *V_ext_*. (**b**) Dependence of voltage amplification factor *R_V_* on driving voltage *V_ext_*. In the experiment, *f_ext_* = 85 Hz. The hot heat exchanger is heated by heating rods, and the cold heat exchanger is cooled by the circulating water.

Following the study of excitation frequency, the influence of driving voltage *V_ext_* is examined. Figure 9a illustrates the dependence of *R_p_* on *V_ext_* at an excitation frequency *f_ext_* of 85 Hz. The heat input *Q_in_* and water flow rate *Q_out_* are fixed at 35.5 W and 800 L/H, respectively. In the low-voltage range (approximately 2.5–4.0 V), *R_p_* increases progressively with increasing *V_ext_* and reaches a maximum value of about 3.73 near 3.5 V, indicating that a moderate *V_ext_* is conducive to the amplification of acoustic oscillations. As *V_ext_* is further increased, *R_p_* decreases markedly, dropping to approximately 2.7 at *V_ext_* = 8.5 V. This trend suggests that larger driving voltages are not beneficial for acoustic amplification, possibly due to impedance mismatch, additional dissipative losses, or enhanced nonlinear energy dissipation within the system. Figure 9b presents the dependence of *R_V_* on *V_ext_*. The overall trend of *R_V_* is consistent with that of *R_p_*, with a maximum value of approximately 1.15 at *V_ext_* = 3.5 V. As *V_ext_* continues to increase, *R_V_* gradually decreases, reaching about 1.12 at 8.5 V, indicating that the electrical output becomes constrained at higher excitation levels.

Figure 9 shows that under a fixed excitation frequency, both the acoustic oscillations and open-circuit voltage exhibit pronounced non-monotonic dependence on the driving voltage. An intermediate driving voltage (approximately 3.5 V) enables the simultaneous optimization of acoustic amplification and open-circuit voltage, whereas excessively low or high driving voltages are detrimental to overall system performance. These findings provide important guidance for the rational selection of excitation parameters and for achieving stable and efficient system operation.

## 6. Conclusions

Conventional acoustic energy harvesters typically enhance the energy harvesting performance through the optimization of acoustic structures. In contrast, this study explores a novel approach that exploits the thermoacoustic effect to amplify the pressure oscillations within an acoustic energy harvester integrated with a PVDF-based piezoelectric nanogenerator and thereby improving the electrical output. First, a prototype of the acoustic energy harvester was constructed. Its acoustic characteristics were systematically analyzed and the feasibility of thermoacoustic amplification was experimentally validated. Subsequently, the effects of excitation frequency and driving voltage on the performance of the acoustic energy harvester were examined. The main conclusions can be summarized as follows:

(1) Both the acoustic pressure within the acoustic energy harvester and the open-circuit voltage of the PVDF-based piezoelectric nanogenerator increase progressively as the temperature difference across the stack increases, demonstrating that the thermoacoustic effect can be effectively utilized to amplify acoustic oscillations and enhance electrical output.
(2) The excitation frequency of the loudspeaker has a pronounced influence on both the acoustic and electrical characteristics of the acoustic energy harvester. At a fixed driving voltage, varying the excitation frequency reveals distinct frequency bands in which the acoustic oscillations are either amplified or suppressed. The corresponding frequency ranges for voltage amplification or suppression differ slightly from those associated with acoustic pressure.
(3) An optimal driving voltage exists for the enhancement of acoustic energy harvesting. At a fixed excitation frequency, variations in the driving voltage show that the pressure and voltage amplification factors exhibit similar trends. When the driving voltage is 3.5 V, the maximum pressure amplification factor reaches 3.73, while the maximum voltage amplification factor attains 1.15.

To conclude, this study innovatively employs the thermoacoustic effect to amplify acoustic oscillations within a resonator, providing a new pathway for improving the performance of acoustic energy harvesters and introducing a novel application scenario for thermoacoustic technology. In future work, more detailed studies will be conducted on the characterization of the PVDF-based piezoelectric nanogenerator, such as current–voltage (I-V) diagrams [33], instantaneous power at varying resistive loads, and integration with energy storage and power management circuits. In addition, multiple thermoacoustic cores will be connected in series to form thermoacoustic metamaterials [34], with the aim of further amplifying acoustic oscillations, increasing voltage output, and exploring the potential of thermoacoustic metamaterials in energy harvesting applications.

## Figures and Tables

**Table 1:** Key geometrical parameters of the acoustic energy harvester with thermoacoustic core.

Components	Parameters	Values	Components	Parameters	Values
**Acoustic resonator 1**	Inner diameter *D_H_*	0.07 m	**Acoustic resonator 2**	Inner diameter *D_R_*	0.07 m
	Length *L_H_*	0.25 m		Length *L_R_*	0.5 m
	Thickness *t_H_*	0.005		Thickness *t_R_*	
**Heat exchangers**	Diameter *D_HX_*	0.07 m	**Stack with square pores**	Diameter *D_S_*	0.07 m
	Length *L_HX_*	0.008 m		Length *L_S_*	0.02 m
	Porosity *σ_HX_*	0.42		Porosity *σ_S_*	0.85
	Width of grooves *d*	3 mm		Square length *a*	1.8 mm

**Figure A1:**
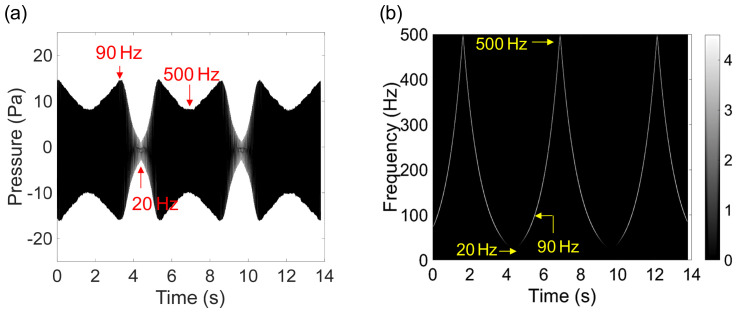
Standalone frequency response of the loudspeaker. (**a**) Acoustic pressure signal. (**b**) Continuous wavelet transform.

## Data Availability

The original contributions presented in this study are included in the article. Further inquiries can be directed to the corresponding author.

## Appendix A. Frequency Response of the Loudspeaker

The frequency response of the loudspeaker operating independently was characterized, as illustrated in Figure A1. To ensure experimental consistency, the voltage amplitude, initial and terminal frequencies, and sweep period of the sinusoidal input signal were maintained identical to those employed in Figure 5. The acoustic pressure signal in Figure A1a was measured with a microphone positioned approximately 2 cm from the loudspeaker diaphragm and its corresponding continuous wavelet transform (CWT) is presented in Figure A1b. The results demonstrate that the sound pressure level varies as a function of excitation frequency, peaking near 90 Hz while attenuating significantly at lower frequencies (e.g., ~20 Hz). This behavior is attributed to the frequency-dependent inductive reactance of the coil, which modulates the current amplitude and, consequently, the acoustic power output. However, the maximum acoustic pressure generated during this standalone test remained below 15 Pa, which is substantially smaller than the peak pressures observed at 79 Hz, 169 Hz, and 316 Hz in Figure 5. The comparison indicates that these three peaks in Figure 5 correspond to the intrinsic resonance frequencies of the acoustic energy harvester rather than the resonance frequencies of the loudspeaker.
